# Editorial: Interaction between automated vehicles and other road users

**DOI:** 10.3389/frobt.2023.1228093

**Published:** 2023-06-16

**Authors:** Philipp Wintersberger, Debargha Dey, Andreas Löcken

**Affiliations:** ^1^ Digital Media Department, University of Applied Sciences Upper Austria, Hagenberg, Austria; ^2^ Visual Computing and Human-Centered Technology, TU Wien, Vienna, Austria; ^3^ Department of Industrial Design, TU Eindhoven, Eindhoven, Netherlands; ^4^ Technische Hochschule Ingolstadt (THI), Ingolstadt, Germany

**Keywords:** automated driving, vulnerable road user (VRU), traffic safety, external human-machine interface (eHMI), human-computer interaction

## 1 Summary

An increasing number of automated vehicles will pervade our traffic systems in the future. The absence of a human driver requires these vehicles to communicate and interact with other traffic participants, such as vulnerable road users (VRUs; pedestrians, cyclists, and emerging mobility forms like eBikes or scooters) or drivers of manual vehicles. In this regard, various studies and concepts demonstrating so-called “external Human-Machine Interfaces” (eHMIs) have been presented in the past couple of years. Many of these works have investigated comparably simple scenarios, such as a single pedestrian aiming to cross the street when an automated vehicle is approaching. In the future, research in this area will have to take more complex situations into account. This drives the need for research addressing other situations involving groups of vulnerable road users and traffic participants, different demographics with different accessibility needs, and different scenarios including roundabouts or urban shared spaces, but also exploring the potential of communication and interaction beyond such classical situations to improve cooperation in traffic.

It is critical to contribute to a more systematic investigation of such communication and interaction systems while providing a forum for thought-provoking ideas and concepts on how automated vehicles and “Internet of Things” (IoT) technology can be utilized to increase safety, cooperation, comfort, empathy, and understanding between a wide range of traffic participants.

This Research Topic aims to address the before-mentioned aspects, but also goes beyond by asking questions like: What does ideal communication between traffic participants look like? What characterizes “good” interaction in traffic? Which ideas and principles should guide communication in the future? Are we just eliminating current problems, or are we ready to develop as-yet-uncovered ideas that may shape interaction in the future?

Within this Research Topic, nine articles have been accepted, which are briefly introduced in the following:

•Fabricius et al. discuss interactions between VRUs and heavy trucks. The authors present a systematic literature review of studies addressing empirical research on the interaction between heavy ground vehicles and VRUs and propose to conduct additional studies to get a deeper understanding of such interactions.
•Loew et al. present the results of a eHMI study in real-world crossing situations. Using the wizard-of-oz method, the authors compared three different eHMI concepts to a baseline and found that all eHMI concepts were rated positively regarding acceptance and perceived safety.
•Zhang et al. studied the interaction between right-turning motorists and crossing cyclists at a traffic-light-controlled urban intersection and identified three common communication patterns. Their results provide insights for implementing a communication strategy for automated driving functions that contributes to both traffic efficiency and ensuring safety when interacting with vulnerable road users.
•Hoggenmueller et al. report on the design and evaluation of an eHMI for a real AV in a pedestrianized urban space. The work presents insights from a human-centered design process and results of s study in virtual reality. The authors argue that the design of eHMIs in complex mobility scenarios requires a more holistic approach.
•Hensch et al. compared 19 younger and 17 elderly peoples’ impressions of eHMIs. In their study, participants experience both well-working and malfunctioning eHMI systems. The authors report that elderly participants assessed eHMIs more positive than younger participants. The authors argue that designing understandable eHMIs demands addressing the requirements of specific user groups.
•Tran et al. present novel wearable augmented reality concepts to assist pedestrians in scenarios where multiple automated vehicles (AVs) travel the road from both directions. The authors evaluated these concepts in a virtual reality experiment. Their results show that wearable AR may reduce pedestrian cognitive load by providing individual AV responses and a clear signal to cross. However, pedestrians’ willingness to adopt a wearable AR solution depends on various factors.
•Lau et al. investigated how the interplay of vehicle kinematics and eHMIs affects pedestrians crossing behavior. They conducted an online study with different eHMI status (static, dynamic, and a baseline) and kinematics (yielding and non-yielding). The results demonstrate that eHMIs can lead to negative effects when not matching vehicle dynamics.
•Sahin et al. present a study conducted in a gamified virtual reality environment, which aimed at revealing how vehicle type, social control, and monetary benefit influences participants’ jaywalking behavior. The results suggest that pedestrians jaywalk more frequently when encountering AVs, and that this behavior is depending on associated risks.
•Mirnig et al. summarize the results of seven studies on eHMIs, which were conducted in three European countries. They discuss the investigation of a great variety of external communication solutions that aim at facilitating the exchange between automated shuttles and other motorized and non-motorized road users.

